# Feasibility and acceptability of a Takeaway Masterclass aimed at encouraging healthier cooking practices and menu options in takeaway food outlets

**DOI:** 10.1017/S1368980019000648

**Published:** 2019-08

**Authors:** Frances Hillier-Brown, Scott Lloyd, Louise Muhammad, Carolyn Summerbell, Louis Goffe, Natalie Hildred, Jean Adams, Linda Penn, Wendy Wrieden, Martin White, Amelia Lake, Helen Moore, Charles Abraham, Ashley Adamson, Vera Araújo-Soares

**Affiliations:** 1 Fuse – UKCRC Centre for Translational Research in Public Health, UK; 2 Department of Sport and Exercise Sciences, 42 Old Elvet, Durham University, Durham DH1 3HN, UK; 3 Redcar & Cleveland Borough Council, Redcar, UK; 4 School of Health and Social Care, Teesside University, Middlesbrough, UK; 5 Kirklees Council, Huddersfield, UK; 6 Institute of Health & Society, Newcastle University, Newcastle upon Tyne, UK; 7 Human Nutrition Research Centre, Institute of Health & Society, Newcastle University, Newcastle upon Tyne, UK; 8 UKCRC Centre for Diet and Activity Research (CEDAR), MRC Epidemiology Unit, University of Cambridge, Cambridge, UK; 9 School of Science, Engineering & Design, Teesside University, Middlesbrough, UK; 10 Research and Innovation Services, Durham University, Durham, UK; 11 Institute of Heath Research, University of Exeter Medical School, Exeter, UK

**Keywords:** Takeaways, Food environments, Public health, Diet, Obesity, Foodscape, Behaviour change

## Abstract

**Objective:**

To evaluate the feasibility and acceptability of the Takeaway Masterclass, a three-hour training session delivered to staff of independent takeaway food outlets that promoted healthy cooking practices and menu options.

**Design:**

A mixed-methods study design. All participating food outlets provided progress feedback at 6 weeks post-intervention. Baseline and 6-week post-intervention observational and self-reported data were collected in half of participating takeaway food outlets.

**Setting:**

North East England.

**Participants:**

Independent takeaway food outlet owners and managers.

**Results:**

Staff from eighteen (10 % of invited) takeaway food outlets attended the training; attendance did not appear to be associated with the level of deprivation of food outlet location. Changes made by staff that required minimal effort or cost to the business were the most likely to be implemented and sustained. Less popular changes included using products that are difficult (or expensive) to source from suppliers, or changes perceived to be unpopular with customers.

**Conclusion:**

The Takeaway Masterclass appears to be a feasible and acceptable intervention for improving cooking practices and menu options in takeaway food outlets for those who attended the training. Further work is required to increase participation and retention and explore effectiveness, paying particular attention to minimising adverse inequality effects.

Improving public health through environmental changes offers a population-wide, preventive approach that reduces the need for individuals to engage directly with interventions^(^^1^^,^^2^^)^. The ‘obesogenic environment’ refers, in part, to the easy availability of highly energy-dense, palatable, inexpensive foods^(^^3^^–^^5^^)^. Many takeaway food outlets contribute to the obesogenic environment. Most takeaway foods are high in fat, salt, sugar and energy density, with average portion sizes often providing a large proportion of, or even exceeding, recommended daily quantities for these nutrients in one meal^(^^6^^,^^7^^)^. Takeaway and fast food consumption has been associated with diets of high energy and poor nutritional quality^(^^8^^–^^11^^)^, as well as with adverse metabolic health outcomes, increased weight and diabetes^(^^11^^–^^16^^)^.

In England, the density of takeaway and fast food outlets increases with area deprivation level^(^^17^^)^. Although there is no strong association between the socio-economic position of an individual and frequency of takeaway and fast food consumption^(^^18^^)^, increased access to takeaway and fast food outlets and differences in consumption patterns of takeaway and fast foods may be leading to increased socio-economic inequalities in dietary intakes^(^^9^^,^^19^^,^^20^^)^.

One approach to reducing access to ‘unhealthy’ takeaway foods is to restrict numbers of new takeaway food outlets opening via the local planning system^(^^21^^–^^24^^)^. A complementary approach is to improve the healthiness of existing takeaway food products. The Department for Health in England has worked with a number of national and regional chain food outlets to promote healthier ready-to-eat meals through the ‘Public Health Responsibility Deal’^(^^25^^)^. Although few independently owned food outlets have signed up to the local equivalent, efforts are being made nationally and internationally to improve the healthiness of existing takeaway food outlet products in a variety of out-of-home food outlets using a range of strategies^(^^26^^,^^27^^)^. Evaluations of local-level interventions, however, are sparse^(^^27^^)^. In 2016, Redcar & Cleveland Borough Council, a local authority in the north-east of England with worse than average deprivation and health^(^^28^^)^, commissioned the delivery of an established training course for staff of independent takeaway food outlets: the Takeaway Masterclass. Health promotion officers from Redcar & Cleveland Borough Council believed the intervention to be a cost-effective way of meeting a health priority need of the local authority and were also keen that some formal evaluation was conducted. The health promotion officers and research team were connected through Fuse, the Centre for Translational Research in Public Health, which aims to close gaps between academia and practice, and find research solutions to address pressing local issues^(^^29^^)^.

The aim of the present work was to explore the feasibility and acceptability of the Takeaway Masterclass and the behaviours it promotes. Assessing feasibility and acceptability is an important, recognised stage in the development and evaluation of complex interventions as potential problems in compliance, intervention delivery, recruitment and retention can be identified^(^^30^^)^. The present work was conducted as part of a larger programme of research that has evaluated a range of interventions aimed at improving the healthiness of takeaway foods^(^^31^^–^^33^^)^. The work demonstrates a co-production (public health practitioners and academics) approach to evaluating real-world, local-level public health programmes that produces practice-based evidence and reduces barriers to knowledge translation^(^^34^^,^^35^^)^.

## Materials and methods

### Intervention description

The Takeaway Masterclass is a three-hour training course delivered by public health professionals and an industry expert to staff from independent takeaway food outlets to promote healthier changes to cooking practices and menu options. The training is designed to be interactive with a mix of information provision (nutrition and cooking skills education), practical activities (taste testing, sugar estimation) and cognitive-change techniques including goal setting and action planning. The training was developed by the Kirklees Food Initiatives and Nutrition Education (FINE) Project health improvement team from Kirklees Council, a local authority in the Yorkshire and Humber region of England, who had previously delivered it seven times over a two-year period in that region^(^^27^^)^. This version of this Takeaway Masterclass was evaluated in-house by the FINE team in terms of recruitment and reach, and feedback on the day of the training. Results are available on request from the FINE team as grey literature reports, and are also summarised in a systematic mapping review^(^^27^^)^.

The Takeaway Masterclass evaluated in the present study was delivered by the same health improvement team but was adapted from the original format. Adaptations included incorporation of updated information on obesity and health and the addition of enhanced goal-setting and action-planning activities developed by members of the research team with behaviour change expertise. A description of the Takeaway Masterclass using the template for intervention description and replication (TIDieR) guidance for reporting interventions^(^^36^^)^, the intervention logic model defined by the research team and the intervention materials are provided in the online supplementary material (Supplemental Table 1 and Supplemental Figs 1–4).

Owners and managers of takeaway food outlets with a hygiene rating of 3 or above (as assessed by a food safety officer as part of England’s Food Hygiene Rating scheme^(^^37^^)^; scores range from 0 (urgent improvement is required) to 5 (hygiene standards are very good)), located in or near to the border of Redcar and Cleveland, were invited to attend a Takeaway Masterclass session. They were given the choice of one of two sessions held on the same weekday (morning or afternoon). Invitations were sent six weeks prior to the event by post. Leaflets advertising the sessions were also distributed by the local authority public health team to eligible food outlets.

The behaviours promoted during the Takeaway Masterclass were based on seven nutrition categories: (i) reducing sugar; (ii) reducing salt; (iii) reducing fat; (iv) increasing fruit and vegetables; (v) increasing fibre (additional to increasing fruit and vegetables); (vi) reducing portion size; and (vii) adding healthier meal deal options. Using the Nuffield intervention ladder^(^^38^^)^, the behaviours promoted included those that restrict choice (e.g. changing oil management practices so all food is cooked in ‘healthier’, better-quality oil; reducing fat, salt and/or sugar in recipes as standard), guide choice through incentivisation (e.g. providing meal deals with healthier options) and enable choice (e.g. the provision of healthier menu options alongside regular options or offering different portion sizes). No information provision type changes (e.g. nutritional labelling) were encouraged during the training, and no instructions or recommendations were given as to whether owners or managers should publicise changes that eliminate choice and may not be noticed by the customer (e.g. changing oil management practices; changing recipes).

### Study design

The present feasibility and acceptability study used a mixed-methods approach with uncontrolled before-and-after and cross-sectional elements. The data collection activities were conducted by members of the research team not involved in the intervention development. Two data collection methods were tested for feasibility and acceptability: (i) relatively burdensome face-to-face assessment visits collecting baseline and follow-up quantitative data (including a secret shopper element) and follow-up qualitative data (‘before and after’ condition); and (ii) less burdensome follow-up qualitative data collection only via telephone (‘follow-up’ condition). Two weeks prior to the Takeaway Masterclass event, researchers purposively assigned the food outlets whose owners or managers had signed up to the Takeaway Masterclass to each group, to ensure maximum variation with respect to type of takeaway food outlet (e.g. fish and chip, Chinese, pizza) and area-level deprivation. The Index of Multiple Deprivation (IMD)^(^^39^^)^ score of the area in which the food outlet was located was used as the measure of area-level deprivation. The most deprived areas were classified as those with an IMD score in the lowest quintile.

**Fig. 1:**
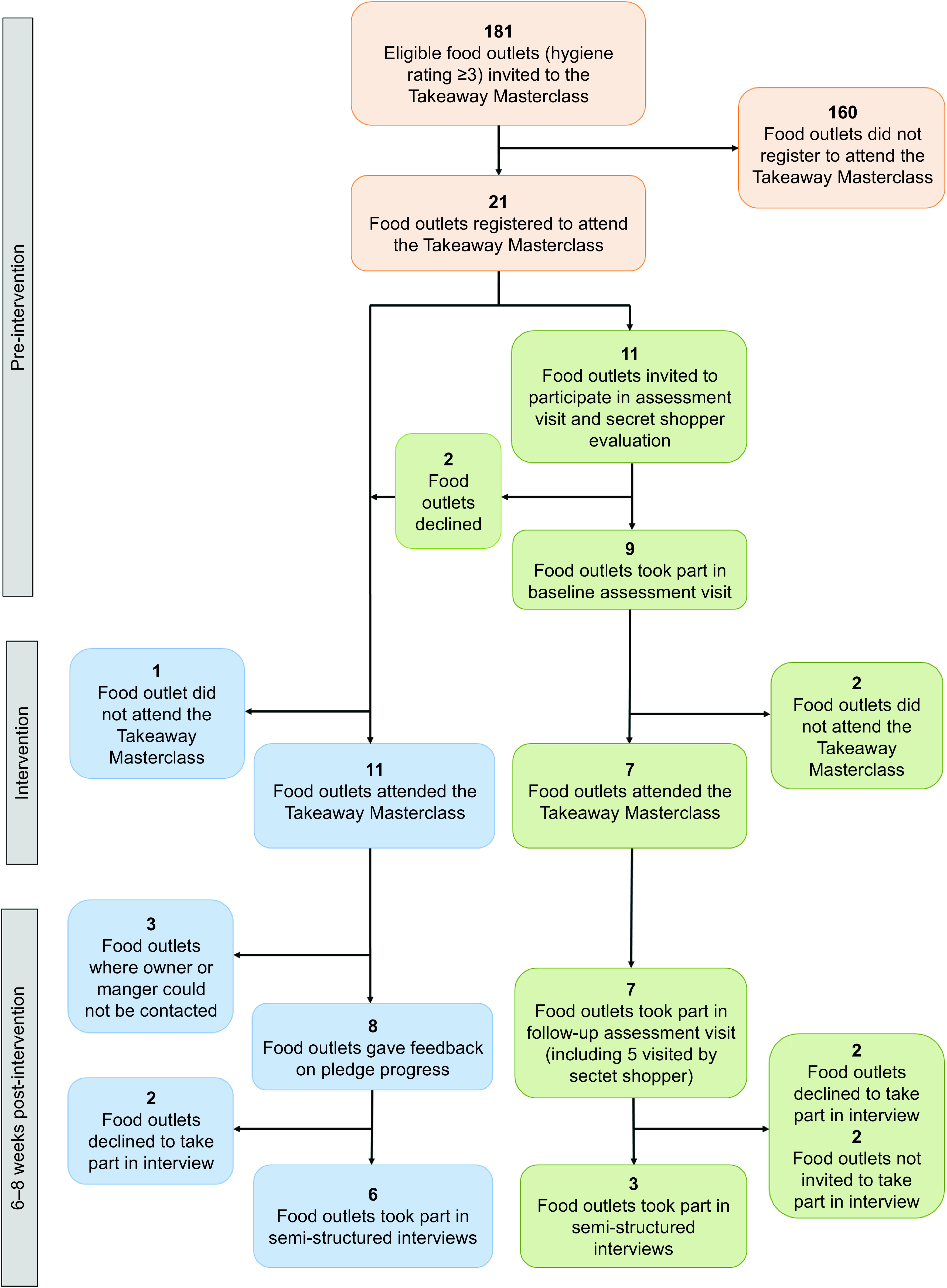
Takeaway Masterclass intervention and evaluation participation flowchart

Owners or managers of food outlets in the ‘before and after’ condition were invited to participate in baseline and follow-up data collection by the research team. During the Takeaway Masterclass event, the remaining owners and managers who attended were asked to give permission for the research team to contact them in six weeks’ time and ask for feedback on their pledge progress. A study flowchart is presented in Fig. 1.

### Consent and ethical approval

The study was conducted according to the guidelines laid down in the Declaration of Helsinki and all procedures involving human subjects were approved by the Newcastle University Research Ethics Committee (reference 5519/2016). All participants provided written informed consent prior to participation.

### Data collection

In the ‘before and after’ food outlets, assessments of catering practices and menu options were conducted by a researcher one to two weeks prior to the intervention and again six to eight weeks after the intervention using a predefined checklist during a visit to the outlet. The checklist included the health-promoting practices that were endorsed during the Takeaway Masterclass (e.g. reducing salt, improving frying techniques, using healthier frying oil, etc.) as well as criteria for the Chartered Institute of Environmental Health’s Healthy Catering Commitment^(^^40^^)^. These health-promoting practices are listed in Table 2 and are classified using the Typology of Interventions in Proximal and Physical Micro-environments (TIPPME)^(^^41^^)^ in the online supplementary material, Supplemental Table 2. Food outlets were assessed on whether they were adhering to each practice or not, or whether it was not applicable to the food outlet (e.g. the checklist item ‘Where sandwiches served, at least two lower-fat fillings are available’ was not applicable to food outlets that did not serve sandwiches). Many of the practices relied on self-reported data from the food outlet owners or managers, although some could be confirmed visually during the visits or determined from the food outlet’s menu.

During the Takeaway Masterclass event, a paper copy of the goal or goals made by all food outlet owners and managers who attended was retained by the research team. At the follow-up visits in the ‘before and after’ food outlets, before the researcher completed the checklist, owners or managers were asked if they achieved their goals or made any other changes. In addition, at approximately six weeks after the intervention, a different member of the research team (unknown to the food outlet) also visited the sub-sample of food outlets as a customer (‘secret shopper’) and completed a modified version of the checklist that included only items related to the specific goals made for the food outlet and other observable items that a customer may be able to identify. Owners or managers from all the ‘follow-up’ food outlets who attended the Takeaway Masterclass training were contacted at six to eight weeks post-intervention via telephone by the research team to ask if they had achieved their goals or made any other changes.

In both groups, during the follow-up visits or phone calls, the owners or managers were invited to take part in a 30 min semi-structured interview with a researcher to further explore the experience of taking part in the intervention and evaluation activities. All semi-structured interviews were audio-recorded and transcribed verbatim.

### Data analysis

Frequency counts were used to analyse the quantitative data to explore feasibility and acceptability and provide estimates for future work. The interview transcripts were read by one researcher to identify emergent and recurrent themes, which were checked by a second researcher. Interview transcripts were analysed using Burnard’s systematic thematic content analysis^(^^42^^)^. This method is an adaptation of grounded theory incorporating thematic and content analysis for a systematic approach to qualitative data analysis in order to reduce researcher bias and increase the reliability of the analysis.

## Results

### Recruitment and retention

A total of 181 takeaway food outlets were invited to attend a Takeaway Masterclass session. Of these, twenty-one (12 %) registered for one of the sessions (Fig. 1). Representatives from three of these food outlets were unable to attend on the day of the Takeaway Masterclass (one because of staffing issues, reasons unknown for the other two), leaving eighteen food outlets (10 % of all invited) attending in total. The proportion of food outlets in the most deprived IMD quintile that attended the event (Table 1) was similar to the proportion of all eligible food outlets located in the most deprived quintile (60 %; data not shown) that were invited to attend.

**Table 1:** Characteristics of the takeaway food outlets participating in the Takeaway Masterclass and evaluation activities, North East England, April 2016

	Baseline*		Attended Masterclass						6-week follow-up						Semi-structured interviews					
	BA group		BA group		FU group		All		BA group		FU group		All		BA group		FU group		All	
	*n*	%	*n*	%	*n*	%	*n*	%	*n*	%	*n*	%	*n*	%	*n*	%	*n*	%	*n*	%
Food outlet type																				
Fish and chips	2	22	1	14	3	27	4	22	1	14	2	25	3	20	1	33	2	33	3	33
Pizza, burger and kebab	5	56	4	57	5	45	9	50	4	57	3	38	7	47	2	67	2	33	4	44
Chinese	0	0	–		1	9	1	6	–		1	13	1	7	–		0	0	0	0
Indian	1	11	1	14	0	0	1	6	1	14	–		1	7	0	0	–		0	0
Café/sandwich	0	0	–		2	18	2	11	–		2	25	2	13	–		2	33	2	22
Multi-cuisine	1	11	1	14	0	0	1	6	1	14	0	0	1	7	0	0	0	0	0	0
Total *n*	9		7		11		18		7		8		15		3		6		9	
IMD quintile																				
1 (most deprived)	3	33	3	43	8	72	11	61	3	43	5	63	8	53	1	33	1	17	2	22
2–5	6	67	4	57	3	27	7	39	4	57	3	38	7	47	2	67	5	83	7	78
Total *n*	9		7		11		18		7		8		15		3		6		9	

BA, before and after; FU, follow-up; IMD, Index of Multiple Deprivation.

* Two food outlets (Chinese (IMD quintile 1) and pizza, burger and kebab (IMD quintile 1)) declined to take part in BA group but did attend Masterclass and so joined FU group.

Retention to the goal attainment follow-up data collection was just over 80 %, and none of the food outlets taking part in the assessment visits that attended the training dropped out at follow-up (Fig. 1 and Table 1). Recruitment to the semi-structured interviews was less successful (Fig. 1 and Table 1). Four who declined had all taken part in both assessment visits and gave reasons of participation fatigue, and two were not invited to take part because of language barriers (a translator for non-English speakers was not available for use in the present study).

Retention rates across the evaluation activities were examined by subgroup of food outlets by deprivation level of the area in which they are located. Only food outlets in the most deprived areas dropped out of the study; two at the goal attainment stage and a further six did not complete a semi-structured interview (see Table 1).

### Goal setting and progress

Each manager or owner of the eighteen food outlets attending the Takeaway Masterclass committed to at least one goal to make a health-benefiting change to their usual practice. The number of goals committed to ranged from 1 to 7, with a median of 4 goals per food outlet, and a total of 69 goals overall (see online supplementary material, Supplemental Table 3). Goals targeting the reduction of salt, sugar and fat were the most popular.

Feedback received at the follow-up contact indicated that a median of 3 goals (range 1–6) per food outlet were achieved (74 % of total goals set). Goals that were reportedly achieved were based on changing ingredients during cooking (e.g. salt and sugar in pizza dough), increasing salad portions and adding more vegetables to meals, changing cooking practices (grilling and poaching), and stocking water and/or reduced-sugar drinks. Changing to lower-fat milk (mainly as an ingredient used to make béchamel sauce of a popular dish traditional to the local area) had mixed success, with some owners and managers commenting that customers had noticed a difference in taste and provided negative feedback, while others stated customers had not noticed, or preferred, the change. Goals that were not achieved were those based on the use of products with reduced salt and sugar (e.g. tomato ketchup and baked beans) because owners or managers were not able to source these products from regular suppliers and/or at reasonable price. Goals based on the use of wholemeal flour products were introduced but not maintained because customers did not purchase these products.

The median number of goals set for the food outlets was 4 (range 1–5) in the ‘before and after’ condition and 4⋅5 (range 1–7) in the ‘follow-up’ condition. A median of 2 (range 1–4) goals, 70 % of goals set, were achieved in the ‘before and after’ food outlets, while a median of 3⋅5 (range 1–6) goals, 77 % of goals set, were achieved in the ‘follow-up’ food outlets.

### Assessment visits (sub-sample)

The number of healthier changes in practice achieved in food outlets at follow-up is displayed in Table 2. A number of these recommendations were already routine practice in most of the food outlets at baseline, including: selling low-sugar drinks; using straight-cut chips rather than crinkle-cut; allowing customers to add their own salt, but not having salt cellars on display (customers needed to request the use of salt cellar); including salad as a side option on their menu and providing salad with meals; using vegetable oil for frying and cooking; and using lean meats and removing fat from meats. At baseline, none of the food outlets sold fresh fruit or fruit juice; incorporated healthy options as part of meal deals; used reduced-fat sauces (e.g. light mayonnaise) or spreads; or used thick-cut chips. At follow-up, the most common changes were reducing the quantity of salt in cooking and making a change to oil management practices (e.g. refilling and filtering more regularly, or using better-quality oil). Only eight positive changes observed from baseline to follow-up were associated with a goal set for a food outlet. The remaining fourteen goals that were specific to behaviours on the assessment checklist were not achieved. The remaining changes that were made (*n* 16) were achieved without a goal having been set.

**Table 2:** Number of takeaway food outlets participating in the Takeaway Masterclass (*n* 7) that achieved health-promoting practices at baseline and 6–8-week follow-up assessments, and the number of goals set for takeaway food outlets related to the practices assessed that were achieved or not at follow-up, North East England, April 2016

	Number of takeaway food outlets achieving health-promoting practice			Number of goals related to health-promoting practice	
Health-promoting practice	Baseline	Follow-up	Not applicable	Achieved	Not achieved
Reduce saturated fat					
Use vegetable oil rather than lard or ghee OR Polyunsaturated or monounsaturated fat or oil used for cooking	7	7	0		
Polyunsaturated or monounsaturated fat or oil used for preparation	4	4	3		
Excess fat drained from food before serving	7	7	0		
Use a lower-fat cheese alternative e.g. mozzarella or half-fat cheddar	0	1	1	1	0
Low-fat sauces e.g. light mayo	0	0	0	0	1
Use lean meats or mince	6	6	1		
Trim visible fat off meat	6	6	1		
Take the skin off the chicken before cooking	6	6	1		
Low-fat spreads/margarine	0	0	0		
Ensure alternatives to chips are not fried	5	5	0		
If chips are served, there is always a healthier starchy alternative	4	4	0		
Offer thick-cut or steak-cut chips	0	0	0		
Use straight-cut chips rather than crinkle-cut	7	7	0		
Use healthier cooking methods for at least one main menu item e.g. stir frying, poaching, boiling, grilling, dry frying and baking	7	7†	0		
Where rice is served, boiled/steamed is available as an alternative	2	2	5		
Where sandwiches served, at least two lower-fat fillings are available	1	1	6		
Semi-skimmed (or skimmed) milk used in cooking	0	4	3		
Semi-skimmed (or skimmed) milk available for drinks	1	2	0		
Total	63	69	21	1	1
Reduce salt					
Reduce the quantity of salt used in cooking*	–	5	0	1	3
Reduce the quantity of sauce used in cooking e.g. curry sauce, soya sauce*	–	1	0	1	1
Salt not added to water used for cooking vegetables, rice and pasta	4	4	3		
Buy ‘no added salt’ canned vegetables and pulses	2	2	5		
Rinse canned vegetables and pulses in water before use	0	0	7		
Do not have salt cellars out on display	6	6†	1		
Customers can add own salt	6	6†	1		
Reduced-hole salt shaker is used	1	0‡	1		
Use reduced-salt options e.g. gravy, ketchup, soya sauce, baked beans, stock cubes	0	0	0	0	1
Total	19	24	18	2	5
Reduce sugar					
Offer reduced-sugar drink alternative instead of or as well as regular products e.g. diet fizzy drinks, water	7	7†	0	1†,§	0
Offer a reduced-sugar product alternative instead of or as well as regular products e.g. reduced-sugar ketchup, reduced-sugar baked beans	0	1	0	0	2
Reduce sugar used in cooking*	–	2	0	1	0
Use sweeteners as a substitute for sugar	0	0	4		
Lower-sugar snacks are available as alternative to biscuits, chocolate, etc.	0	0	4		
Total	7	10	8	2	2
Increase fruit and vegetables					
Provide a salad garnish with each meal	6	6	1		
Offer salad as an alternative to chips	5	5	1		
Include salad as an option on sides menu	6	6	1		
Include fresh fruit juice as a drink option (150 ml carton)	0	1†	0	0	2
Incorporate more vegetables in recipes where appropriate	–	2	0	2	1
Serve baked beans or sweetcorn as a side (e.g. with fish and chips)	1	1	6		
Fresh fruit is always available and prominently displayed	0	0	0	0	1
Total	18	21	9	2	3
Increase fibre					
Use wholemeal pasta and/or noodles	0	0	5		
Use brown rice	0	0	5	0	1
Use part wholemeal flour in naan	0	0	6		
Use wholemeal bread e.g. rolls, pittas, wraps	1	1	0	0	2†
Total	1	1	16	0	3
Reduce portion size					
Reduce ‘standard’ portion sizes (e.g. portion of chips)*	–	1	0		
Offer a smaller portion size option as well as a regular portion size	3	4†	0		
Smaller portions are available for children	3	3	0		
Total	6	8	0	0	0
Overall health-promoting practices					
Incorporate healthier options as part of meal deals	0	0†	0		
Healthy eating is promoted by staff	1	2	0		
Improve oil management*	–	4	0	1	0
Total	1	6	0	1	0

* Assessment at follow-up only.

† Practice confirmed by researcher acting as a ‘secret shopper’.

‡ Regular salt shaker used for regular salt and reduced-hole salt shaker used for low-sodium salt at follow-up.

§ Reduced-sugar (diet) drinks were already supplied at the food outlet at baseline, but goal was to provide bottled water in addition.

### Secret shopper (sub-sample)

Of the seven food outlets taking part in the follow-up assessment visits, five had set goals that were deemed feasible for observation by a secret shopper. These observations confirmed some of the findings from assessment visits (Table 2). Changes that were easy to observe were menu board items and specials boards, placement of salt shakers, availability and placement of drinks, and provision of particular products (e.g. wholemeal bread buns). It was difficult to determine the type of sauces being provided (e.g. reduced-salt and -sugar tomato ketchup and reduced-fat mayonnaise) as these are often contained in plain sauce bottles, and it was not always possible to observe all the selected changes at the time of the visit.

### Intervention and evaluation acceptability

Data on intervention acceptability, for those who attended the Takeaway Masterclass, were collected through semi-structured interviews with owners and/or mangers (*n* 9). The main themes arising from these data are discussed below.

#### Takeaway Masterclass event acceptability

The Takeaway Masterclass was well received and participants would recommend the event to others. Overall, participants were positive about the venue, the timing and delivery of the event, and the materials provided. There was a small amount of negative feedback: one participant had found the venue difficult to find, and some thought that some of the content was not applicable to them (e.g. the oil management component was primarily applicable to fish and chip food outlets).

#### Direct effects of the Takeaway Masterclass training and effects after making changes

Clear changes in knowledge and opinions were expressed by a number of participants during the semi-structured interviews. Increased nutritional awareness (e.g. quantity of sugar in food items) and awareness of new ideas were stated as benefits of taking part in the training. Participants reported making changes such as reformulating recipes (e.g. reducing the amount of sugar in flapjacks, reducing salt in pizza bases) and providing additional healthier items (e.g. sandwich and salad options, smaller portion options) to their menus to increase the choice available to their customers. Positive feedback from customers had resulted in some changes being sustained and some food outlets trying additional changes. Positive effects from the changes made that were reported included: perceived increases in customers; better quality of products; competitive edge or unique selling point as other food outlets do not offer the same healthier products; financial benefits and increases in sales. Negative effects resulting from making changes were also reported: negative customer feedback; lost business and healthier items not selling. These tended to result in the associated changes being abandoned. However, some owners or managers were happy to use a trial-and-error approach, stating that they would be willing to spend more or sacrifice some loss of business if it was outweighed by increased business overall.

There were some misunderstandings of the messages delivered during the Takeaway Masterclass that became apparent when food outlets were contacted at follow-up as well as during analysis of interview data. These were mainly in relation to the oil management component of the training. Some food outlet owners and managers believed that they needed to change their oil more regularly, when in fact one of the benefits of good oil management is that oil can be changed less regularly. Some food outlet owners and managers also appeared to take on a health-promotion role with an ‘instructing’ or ‘pushy’ style in their interactions with customers – an approach not encouraged by the Takeaway Masterclass training and that was reportedly not well received by customers. Some food outlet owners and managers also stated that they were already implementing some healthier practices (which is supported by the findings from the baseline data collection) and some believed they already had a good level of nutrition knowledge.

#### Barriers and facilitators to making health-promoting changes

Many of the food outlet owners or managers identified fear of negative customer feedback, or receipt of negative feedback, as a barrier to making health-promoting changes to their practices. Many believed that customers would not be receptive to changes or not interested in healthier changes. However, some believed there is customer demand for healthier products, and that positive feedback encouraged changes to be sustained. They reported that changes perceived to affect the taste of a product were unlikely to be tried. Conversely, the perception that a change would not alter the taste of product led to these changes in practice being tried and sustained. Being unable to source certain products, at reasonable prices, and not having suitable equipment were highlighted as barriers to making certain changes, especially for food outlets under financial pressure.

It seems that some changes were acceptable while others were not, and that there was variability in the acceptability of the same change. For example, one owner was happy to change to a lower-fat milk in their béchamel sauce but believed that using a lower-fat alternative to regular butter was a compromise too far. Another food outlet manager, however, believed that using lower-fat alternatives to both whole milk and butter resulted in a better-quality béchamel sauce, preferred by their customers.

The personal beliefs of the food outlet owners and managers around healthy foods and diets appeared to be a major influence in how receptive they were to attempting and sustaining changes. Those owners and managers who appeared most successful in making and sustaining changes stated existing strong personal interests in health and providing healthier food alternatives.

#### Evaluation acceptability

No issues were raised with regard to the evaluation process and all owners and managers were happy with the activities they had been involved in. Some even expressed that they valued the follow-up contact.

## Discussion

### Statement of principal findings

We found evidence that the Takeaway Masterclasses were both feasible and acceptable to those who attended. At least one change in cooking practice or menu options were made in all the food outlets contacted at follow-up, but these were not all changes that were planned during the Takeaway Masterclass. Many changes were verified by observation. The evaluation procedures were largely acceptable.

### Strengths and limitations of the present study

The present feasibility and acceptability evaluation adds to an extremely limited evaluation evidence base of local-level public health interventions that aim to improve the healthiness of foods sold by takeaway food outlets^(^^27^^)^. Although some members of the research team were involved in the modification of the intervention (mainly the goal-setting and action-planning component), these were not members who conducted the evaluation and the majority of the intervention was developed and delivered by local authority public health practitioners. An important strength is that baseline and follow-up measures were piloted in the present study, whereas the majority of the existing evidence base relies on follow-up data only^(^^27^^)^. However, the study did not include control takeaway food outlets, so we were unable to compare outcomes or test the acceptability and feasibility of the evaluation methods in similar outlets not receiving the intervention. Follow-up feedback received by telephone tended to be more positive in the present study, compared with feedback received at face-to-face visits. It was possible to make some objective assessments of practices (direct visual observation and menu information); however, most of outcomes were based on self-reported accounts from the takeaway food outlet owners and managers. The attendees were from a small proportion of eligible food outlets and may not have been representative of the target population. No data were collected on businesses whose manager or owner did not sign up to attend either Takeaway Masterclass session, other than the business name and address, so we were unable to explore reasons for non-attendance. The use of translators should be considered in future work to minimise missing data because of language barriers.

### Strengths and limitations of the intervention

The Takeaway Masterclass is a relatively inexpensive intervention that, after initial development, requires facilitator, room hire and invitation and promotion costs only. The sessions primarily comprised nutritional education delivered by experienced public health practitioners and an industry representative who understood how, and the context in which, takeaway food outlets operate. The training used a mix of educational approaches and motivational techniques in order to maximise engagement with attendees. The practices promoted during the training were mainly coded towards the higher end of the Nuffield intervention ladder and may be more effective than simple information provision^(^^26^^)^. Of course, implementation of the recommended practices depends on the motivation and commitment of individual owners and managers, firstly to attend the training and secondly to implement changes in their food outlet, which often includes the engagement of other staff members. A major barrier to using some of the healthier products promoted during the training was lack of availability (at all or at a price comparable to regular products) from suppliers to the takeaway food outlets. This may a particular problem in the local authority where this intervention took place as takeaway food outlets may have less supplier choice than more populated areas such as major cities in the UK; however, this barrier has been identified in other evaluations of interventions implemented across England^(^^27^^)^.

Of all eligible food outlets, attendance at the two Takeaway Masterclass sessions offered on the one day was 10 %. This is initially higher than seen in the (larger) Kirklees local authority where the training was originally developed and delivered, which took a number of sessions to achieve a 10 % attendance rate; to date engagement has reached 25 % of all eligible takeaway food outlets after nine sessions run over three years (FINE project, unpublished results). This indicates that this type of intervention may only attract a small proportion of the target population at a time and a series of training sessions is needed to increase participation rates. Factors such as providing more flexibility around training timings, days, venue, and possibly language could be explored.

### Interpretation of findings

Previous research has suggested that healthy eating interventions targeting takeaway food outlets may not be taken up by food outlets from more deprived areas^(^^40^^,^^43^^,^^44^^)^. In contrast, the Takeaway Masterclass was attended by owners and managers of food outlets located across the range of deprivation levels in proportion to eligibility. This suggests that the Takeaway Masterclass training event was acceptable to and feasible for food outlets across the deprivation level spectrum. An imbalance in socio-economic status representation was, however, evident at follow-up where a higher dropout rate was observed for food outlets from areas of the highest deprivation level, compared with those from less deprived areas. Care is therefore needed in future intervention delivery and evaluation to ensure strategies to increase retention are in place (e.g. use of translators for participants with limited English language skills). It is possible that those who were more successful in making changes in response to the intervention may have been more willing to provide follow-up data, which could mean that the behaviours promoted at the Takeaway Masterclass may have been more acceptable and feasible in food outlets located in less deprived areas. This could also be a result of the higher economic pressures experienced in the more deprived area.

The barriers and facilitators to making healthier changes to cooking practices and menu options identified in the present work are similar to those found by other projects conducted in England^(^^27^^)^. Changes requiring only a small level of effort at no or little extra cost to the food outlet were the most likely to be implemented and sustained (e.g. reducing current ingredients such as sugar or salt, or changing to healthier products that are easy to source at a similar price to regular products such as using semi-skimmed instead of whole milk). In contrast, using products that were difficult to source at similar prices and quantities to regular products (e.g. reduced-salt and -sugar tomato ketchup) or unpopular with customers (e.g. wholemeal bread) were unlikely to be tried or sustained.

### Unanswered questions and future research

The present study demonstrates the feasibility and acceptability of the Takeaway Masterclass in a small group of takeaway food outlets. Further development and collaboration with stakeholders are needed to identify appropriate intervention delivery methods (timings, venue, language) and outcomes; and the study design for a definitive process, outcome and economic evaluation. Pilot work in a larger sample will allow further testing of data collection methods, ensure confidence that the intervention can be delivered as intended, and allow safe assumptions to be made in terms of effect sizes and variability, and rates of recruitment and retention for a definitive evaluation^(^^30^^)^. There is also the need for work exploring reasons for non-attendance and how participation rates can be increased, and to work with suppliers to increase the availability of affordable healthier products for takeaway food outlets.

## Conclusions

The Takeaway Masterclass appears to be a feasible and acceptable intervention for improving cooking practices and menu options for those food outlets who agreed to take part. However, owners or managers from a relatively small proportion of eligible food outlets attended and may not have been representative of the whole target population. Staff from the takeaway food outlets self-reported making a number of ‘healthy’ changes, but there was minimal objective evidence of change. Further work is required to increase participation and retention and explore effects on menu options and customer purchases, paying particular attention to minimising adverse inequality effects.

## Supplementary material

For supplementary material accompanying this paper visit https://doi.org/10.1017/S1368980019000648. click here to view supplementary material
