# Supplementary material for: Feasibility and acceptability of a Takeaway Masterclass aimed at encouraging healthier cooking practices and menu options in takeaway food outlets
Source: Public Health Nutr. 2019 Aug;22(12):2268–78. doi: 10.1017/S1368980019000648 (PMC6676448; doi:10.1017/S1368980019000648)

**Supplemental Table 1: Description of the Takeaway Masterclass using the template for intervention description and replication (TIDieR) checklist**

| 1. Name | Healthy Takeaway Masterclass |
| --- | --- |
| 1. Rationale, theory, goal | Takeaway food = poor nutrient content and consumption has been linked to weight gain and adverse metabolic outcomes.  The behaviour change techniques used during the training are:   - Providing information on behavioural outcomes - Instructions on how to perform a behaviour. - Demonstration of Behaviour/credible source/ vicarious consequences - Prompt practice/behavioural experiment - SMART goal setting (behaviour) and action planning with problem solving/coping planning - Prompt/cue for action   Aim: Healthier cooking practices are implemented and/or healthier menu options are provided in each takeaway |
| 1. Materials | Information provision on: consequences of behaviour and behaviour change opportunities presented on slides. Using tasting experiences of alternative low sugar, low salt, and low fat ingredients as alternative condiments/produces. Modelling accurate forms of managing frying oil prolonging its quality and life using a video highlighting how to do this step by step. These materials are used during one session (approx. 3 hours). Printed handouts of the slides, a tips booklet on how to increase healthiness of their takeaway produces, a notebook (for goal setting and action planning with problem solving) and a pledge sheet (acting as a prompt/cue for action) are provided to participants to use on the day and take to the business with instructions to place in the kitchen in a easy to look at place. |
| 1. Procedures | Takeaway owners and managers were invited via an official headed letter from the public health team at Redcar & Cleveland (R&C) Borough Council approx. 6 weeks prior to class. Leaflets advertising the training were also distributed in the borough.  On day – training approx. 3 hours that includes: introduction from R&C Health Improvement Commissioning Lead including information on health context nationally and locally; slides providing information on how to increase the healthiness of the food choices offered by the business with real life examples of previous implementations and the benefits of this. This was designed and delivered by three Kirklees Food Initiatives and Nutrition Education (FINE) Project* team members. A video (approximately 7 minutes) with step by step instructions on how to care for frying oil designed, introduced and commented on by one industry representative (National Federation of Fish Fryers [NFFF]); practical activities 1) tasting of regular and ‘healthier’ options (e.g. low vs normal fat mayonnaise) and 2) sugar quantity estimation (in drinks); and goal setting (behaviour) and action planning with problem solving activity. |
| 1. Who provided | Educational materials delivered by three FINE team members and one industry representative (NFFF). Members of the FINE team and R&C Public Health team assisted with the practical sessions. The goal setting and action planning activity was introduced by VAS (Health Psychology, Newcastle University) and support provided by research staff (morning) with FINE and R&C team (afternoon). |
| 1. How | Training delivered in person in a group session |
| 1. Where | Central location in the local authority. Two sessions offered (morning and afternoon) |
| 1. When and how much | One session approx. 3 hours. |
| 1. Tailoring | Businesses of varying type (cuisine) and location attended. Goal setting and action planning was tailored to the business as business set these. |
| 1. Modifications | Modifications to what had previously been delivered in Kirklees: Local context information changed from Kirklees to Redcar and Cleveland; more emphasis made on case study examples; inclusion of video (approximately 7 minutes) on oil filtering; and goal setting with action planning activity significantly enhanced. |
| 1. Planning/fidelity (ways to maintain fidelity) | The same experienced intervention deliverers delivered both sessions, using the same materials. The enhanced goal setting and action planning activity was a new component and the plan was to deliver training to FINE and R&C staff on how to deliver this. |
| 1. Actual fidelity | The parts of the sessions delivered by the FINE team NFFF rep were delivered as intended. It was intended that the enhanced goal setting and action planning activity was also delivered by the FINE team or R&C staff; however, this was introduced and supported by the research team, solely in the morning, and along with FINE and R&C staff in the afternoon. |

**The Kirklees Food Initiatives and Nutrition Education (FINE) Project is a Kirklees Public Health commissioned provider service located within the Environmental Health Department of Kirklees Council. Its main remit is to improve nutrition literacy amongst professionals, staff as well as volunteers. In addition a proportion of their work is to engage in healthy eating interventions with the out of home food sector in Kirklees.*

**Supplemental Table 2: Classification of the health promoting practices evaluated before and after the Takeaway Masterclass using the Typology of Interventions in Proximal and Physical Micro-environments (TIPPME)**

| Health promoting practice | TIPPME classification |
| --- | --- |
| *Reduce saturated fat* | |
| Use vegetable oil rather than lard or ghee OR Polyunsaturated or monounsaturated fat or oil used for cooking | Product, Placement: availability |
| Polyunsaturated or monounsaturated fat or oil used for preparation |  |
| Excess fat drained from food before serving |  |
| Use a lower fat cheese alternative e.g. mozzarella or half fat cheddar |  |
| Low fat sauces e.g. light mayo |  |
| Use lean meats or mince |  |
| Trim visible fat off meat |  |
| Take the skin off the chicken before cooking |  |
| Low fat spreads/margarine |  |
| Ensure alternatives to chips are not fried |  |
| If chips are served, there is always a healthier starchy alternative |  |
| Offer thick cut or steak cut chips |  |
| Use straight cut chips rather than crinkle cut |  |
| Use healthier cooking methods for at least one main menu item e.g. stir frying, poaching, boiling, grilling, dry frying and baking |  |
| Where rice is served, boiled/steamed is available as an alternative |  |
| Where sandwiches served at least two lower fat fillings are available |  |
| Semi-skimmed (or skimmed) milk used in cooking |  |
| Semi-skimmed (or skimmed) milk available for drinks |  |
|  | |
| *Reduce salt* | |
| Reduce the quantity of salt used in cooking^2^ | Product, Placement: availability |
| Reduce the quantity of sauce used in cooking e.g. curry sauce, soy sauce^2^ |  |
| Salt not added to water used for cooking vegetable, rice & pasta |  |
| Buy ‘no added salt’ canned vegetables and pulses |  |
| Rinse canned vegetables and pulses in water before use |  |
| Do not have salt cellars out on display | Related product, Placement: position |
| Customers can add own salt | Product, Properties: functionality |
| Reduced hole salt shaker is used | Related product, Properties: functionality |
| Use reduced salt options e.g. gravy, ketchup, soy sauce, baked beans, stock cubes | Product, Placement: availability |
|  | |
| *Reduce sugar* | |
| Offer reduced sugar drink alternative instead if or as well as regular products e.g. diet fizzy drinks, water | Product, Placement: availability |
| Offer a reduced sugar product alternative instead of or as well as regular products e.g. reduced sugar ketchup, reduced sugar baked beans |  |
| Reduce sugar used in cooking^2^ |  |
| Use sweeteners as a substitute for sugar |  |
| Lower sugar snacks are available as alternative to biscuits, chocolate etc |  |
|  | |
| *Increase fruit and vegetables* | |
| Provide a salad garnish with each meal | Product, Placement: availability |
| Offer salad as an alternative to chips |  |
| Include salad as an option on sides menu |  |
| Include fresh fruit juice as a drink option (150ml carton) |  |
| Incorporate more vegetables in recipes where appropriate |  |
| Serve baked beans or sweetcorn as a side (e.g. with fish and chips) |  |
| Fresh fruit is always available and prominently displayed | Product, Placement: availability and position |
|  | |
| *Increase fibre* | |
| Use wholemeal pasta and/or noodles | Product, Placement: availability |
| Use brown rice |  |
| Use part wholemeal flour in Naan |  |
| Use wholemeal bread e.g. rolls, pittas, wraps |  |
|  | |
| *Reduce portion size* | |
| Reduce ‘standard’ portion sizes (e.g. portion of chips)^2^ | Product, Properties: size |
| Offer a smaller portion size option as well as a regular portion size | Product, Placement: availability |
| Smaller portions are available for children |  |
|  | |
| *Overall health promoting practices* | |
| Incorporate healthier options as part of meal deals | Product, Placement: availability |
| Healthy eating is promoted by staff (*HCC 22) | Wider environment, Properties: information |
| Improve oil management^2^ | Related objects, Properties: functionality |

**Supplemental Table 3: Goals set by owners and managers of the takeaway food outlets**

| 1 | Reduce sugar - change ketchup |  |  |  |  |  |  |
| --- | --- | --- | --- | --- | --- | --- | --- |
| 2 | Swap to lower sodium salt | Buy reduce sugar and salt ketchup | Buy reduced sugar and salt baked beans | Increase fibre - buy brown rolls | Put poached fish on the menu |  |  |
| 3 | Increase fruit and vegetable | Reduce saturated fat |  |  |  |  |  |
| 4 | Add a bigger portion of salad | Remove the salt shaker | Add more healthy drinks to menu | Use less fat products | Refill the oil tank regularly |  |  |
| 5 | Reduce salt (e.g. sauces) | Reduce sugar (Buy sugar free drinks) | Reduce fat (Buy low fat cheese) | Buy brown rice and brown bread buns |  |  |  |
| 6 | Less salt | Low fat mayo | Low sugar tom sauce | Increase veg | Introduce smaller portions |  |  |
| 7 | Reduce salt |  |  |  |  |  |  |
| 8 | Reduce salt and sugar in cooking | Reduced sugar contained drink to water (???) |  |  |  |  |  |
| 9 | Reduce salt | Reduce sugar | Change ??? ??? Oil |  |  |  |  |
| 10 | Offer a fruit and salad option in kid's box | Give option of wholemeal bread bun and pitta's | Reduce the fat in the bechamel sauce | Don't offer to put salt and vinegar to food |  |  |  |
| 11 | Reduce salt in ketchup and beans | Reduce sugar in ketchup and beans | Reduce saturated fat (in fryers) | Use wholemeal pasta/flour | Promote smaller portion sizes more | Include water as part of meal deal | Put water at eye level in fridge |
| 12 | Remove salt from counter | Stock bottled water and more zero sugar | Offer a poached fish | Offer alternative to kid's meal | OAP/child portions(?) | Try some cuscus as an alternative to chips |  |
| 13 | Reduce salt - less hole shakers | Reduce sugar - reduced sugar ketchup |  |  |  |  |  |
| 14 | Reduce sugar and salt on the products | Might add a new salad on menu | Change oil more often | Put on menu healthier products (such as vegetable ???) | Reduce portions on ingredients/meat that contain fats | Change the milk |  |
| 15 | Reduce salt (remove(?) salt shaker) | Reduce sugar (low sugar ketchup) | Healthier option (salad(?)/chips in olive oil) | Reduce fat (green top milk) |  |  |  |
| 16 | Reduce salt - change salt shaker | Reduce sugar - introduce fruit juice | Reduce saturated fat - trim fat from meat | Increase fruit and veg - salad option (instead of just side salad) | Small portion - introduce lite bite | Healthier options - introduce baked potatoes |  |
| 17 | Reduce salt in dough | Recommend water/water on meal deal as opposed to cola | Cucumber sticks with kid's meal | Grilled chicken kid's option | More salad with kebab |  |  |
| 18 | Reduce my sugar by 10-20% in flapjacks/cakes |  |  |  |  |  |  |

**Supplemental** **Fig. 1: Takeaway Masterclass intervention logic model**

INPUT/RESOURCES

ACTIVITY

OUTPUT

SHORT TERM OUTCOMES

MEDIUM TERM OUTCOMES

LONG TERM OUTCOMES

Delivery of interactive workshop, providing training and guidance to takeaway owners/manager to deliver a healthier choice of product

Owner/manager implements one or more of the covert interventions

Owner/manager implements one or more of the overt interventions

Customers accept changes

Customers reject changes

Owner/manager rejects all suggested interventions

Intervention fails

Increased provision of healthier food in takeaway

Decrease in diet related diseases

Healthier diet consumed overall by customers

Decrease in preference for unhealthier foods in customers

More unhealthy foods consumed elsewhere in diet (compensation)

No change in preference for unhealthy foods in customers

PURPOSE: To improve dietary intake of takeaway food customers by increasing the availability of ‘healthier’ food within takeaways

CONTEXT: Regular consumption

**Supplemental Fig. 2: Takeaway Masterclass Notebook**


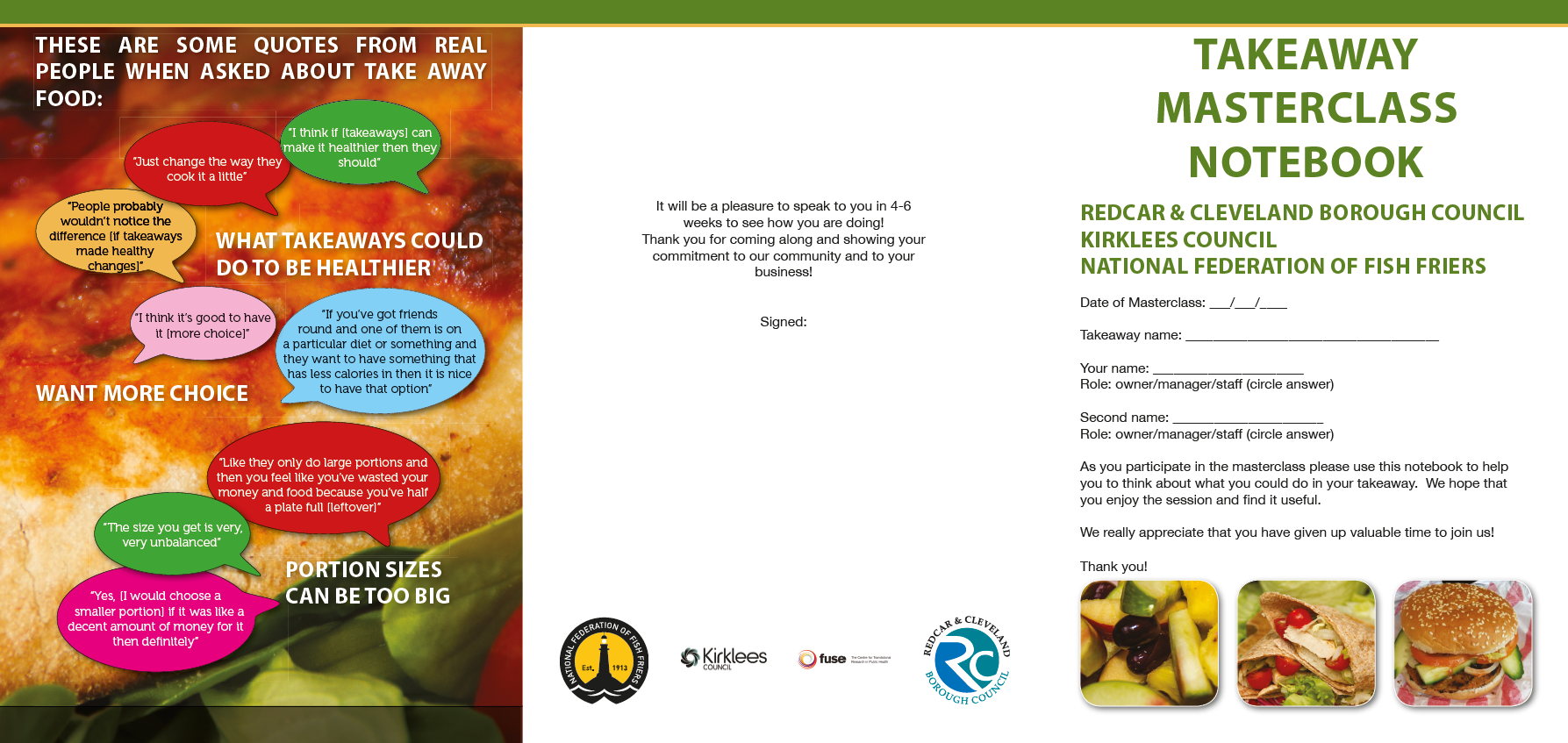


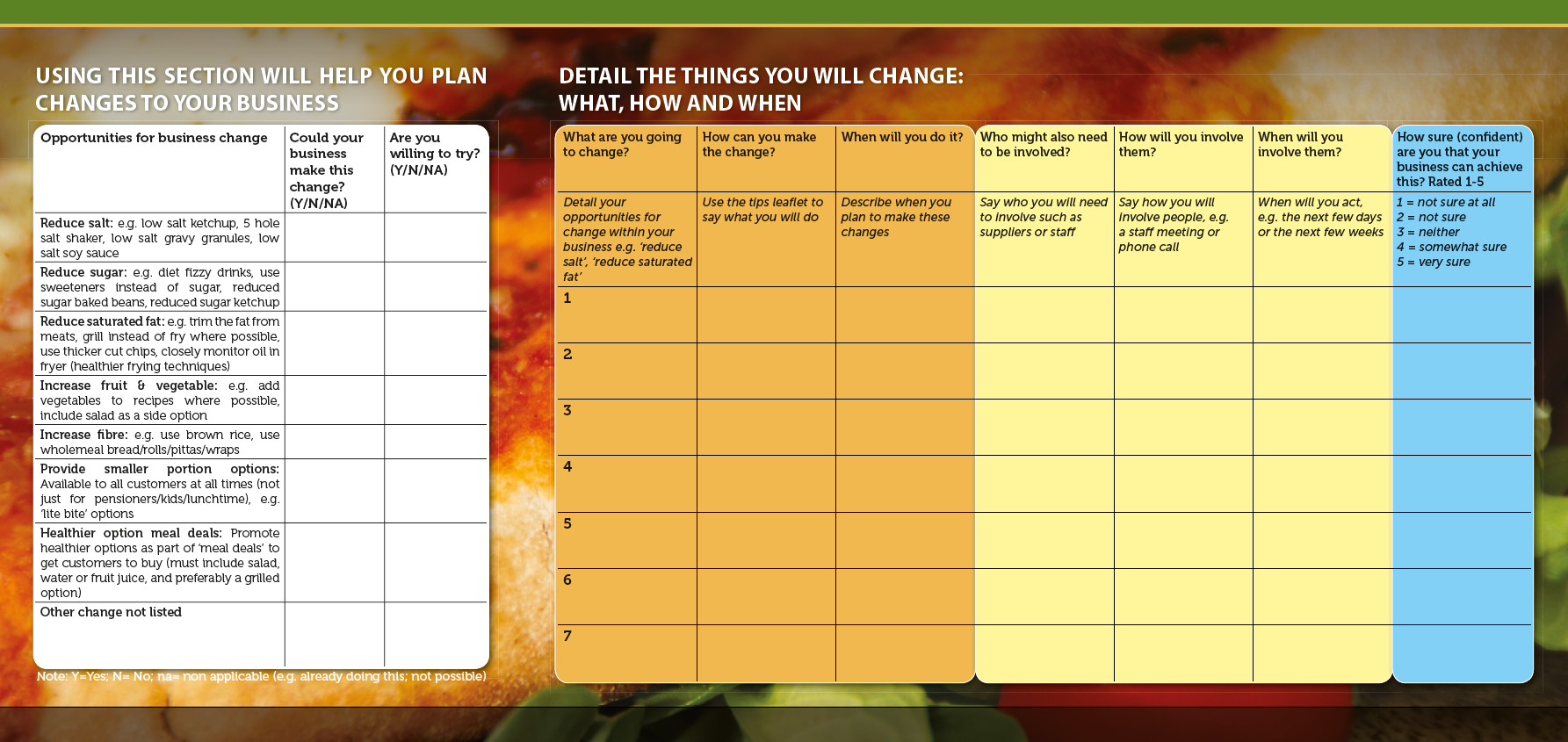


**Supplemental Fig. S3: Takeaway Masterclass Tips Leaflet**


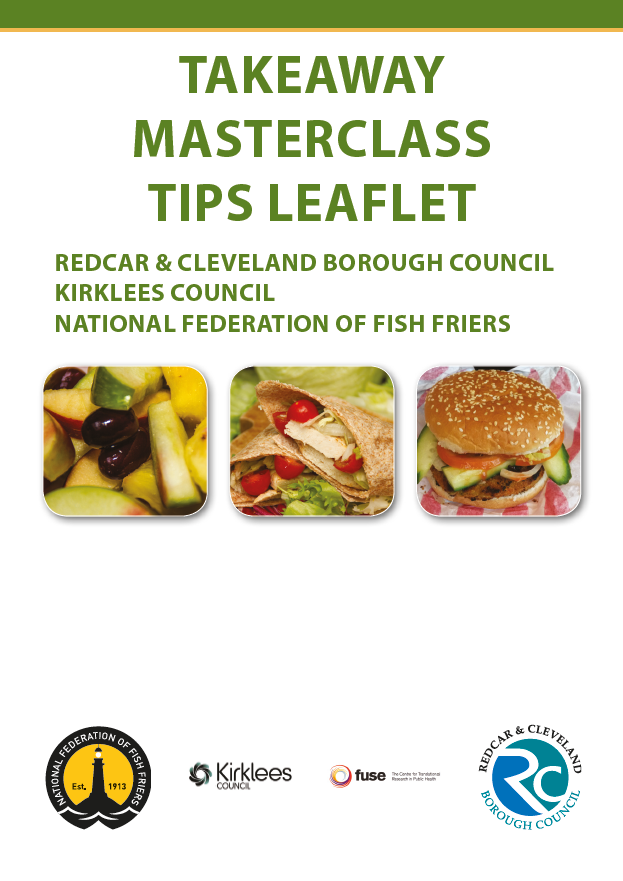

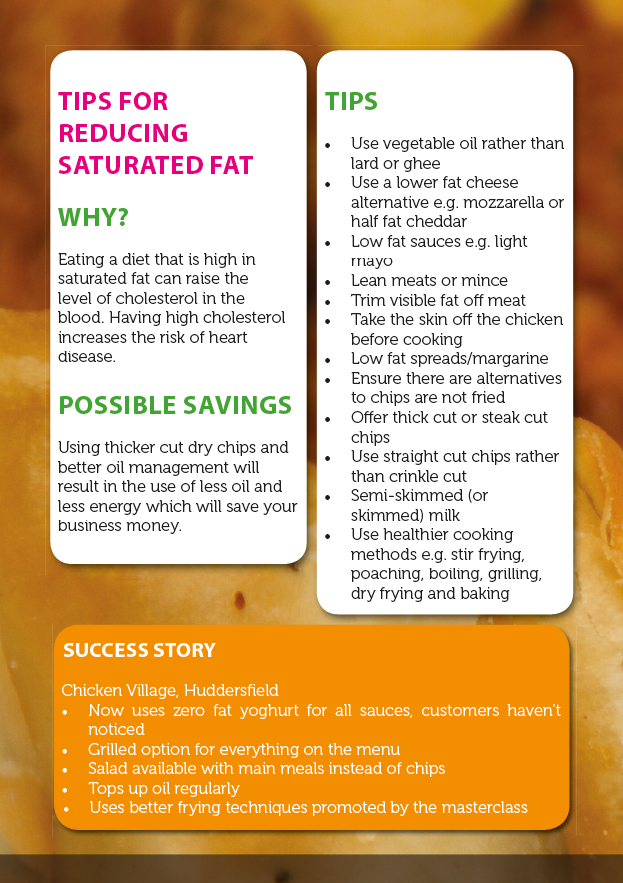


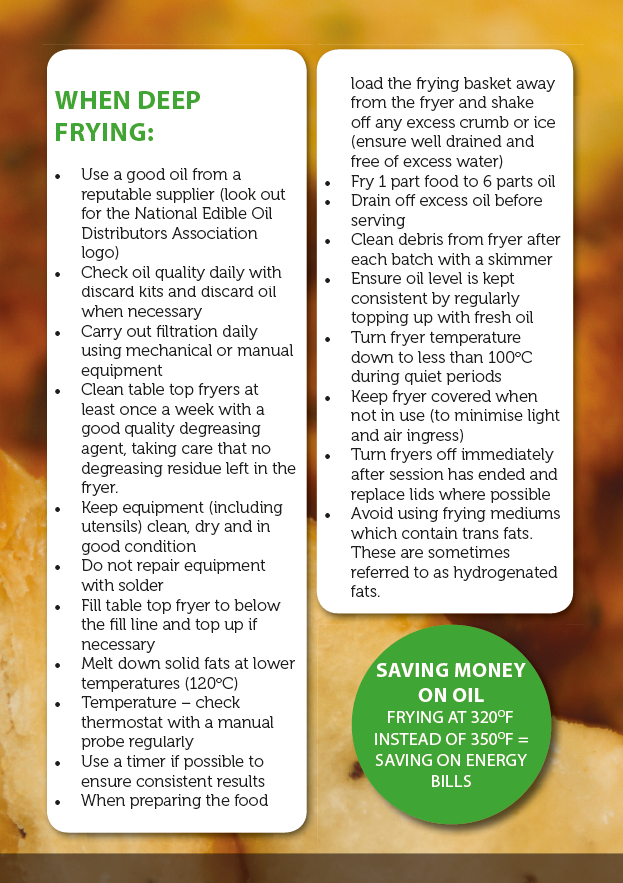

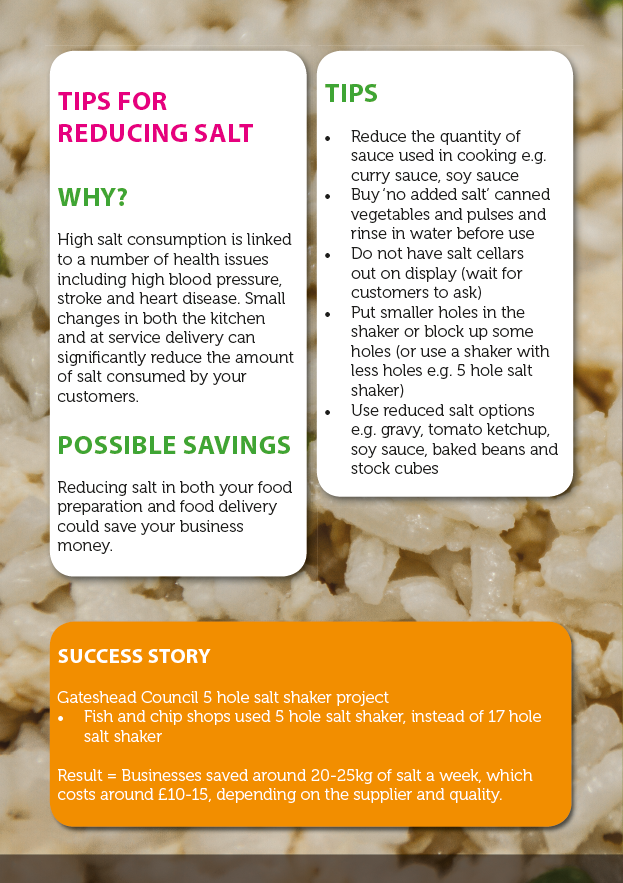


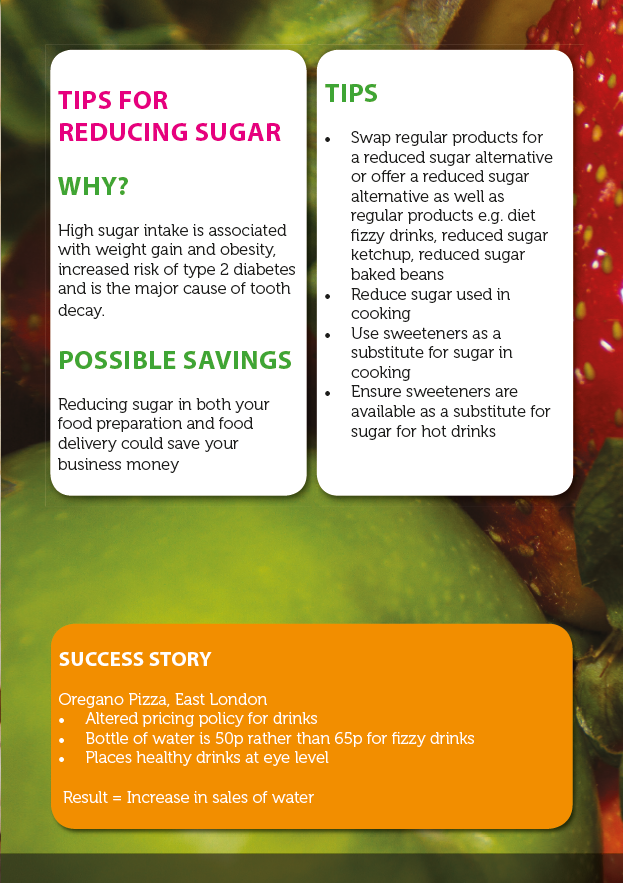

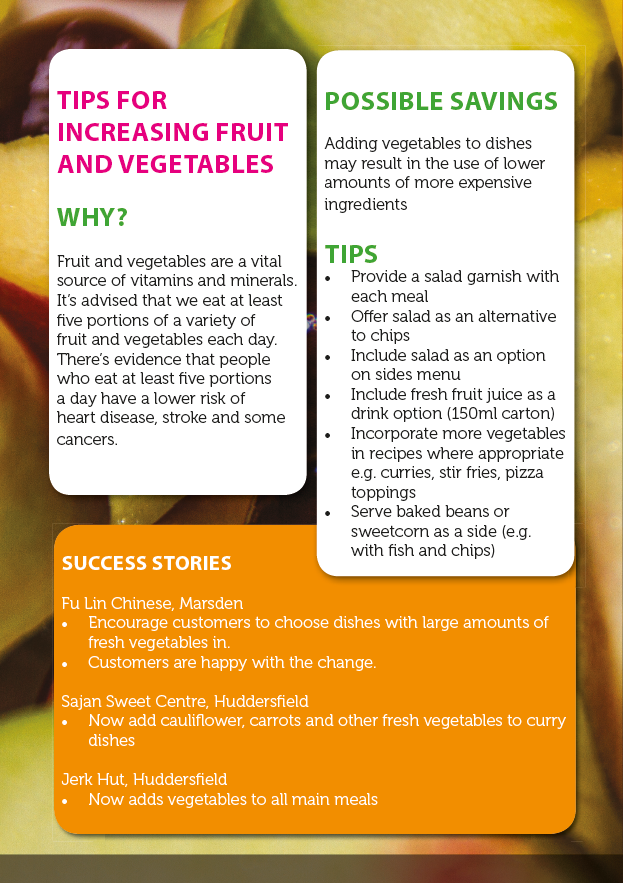


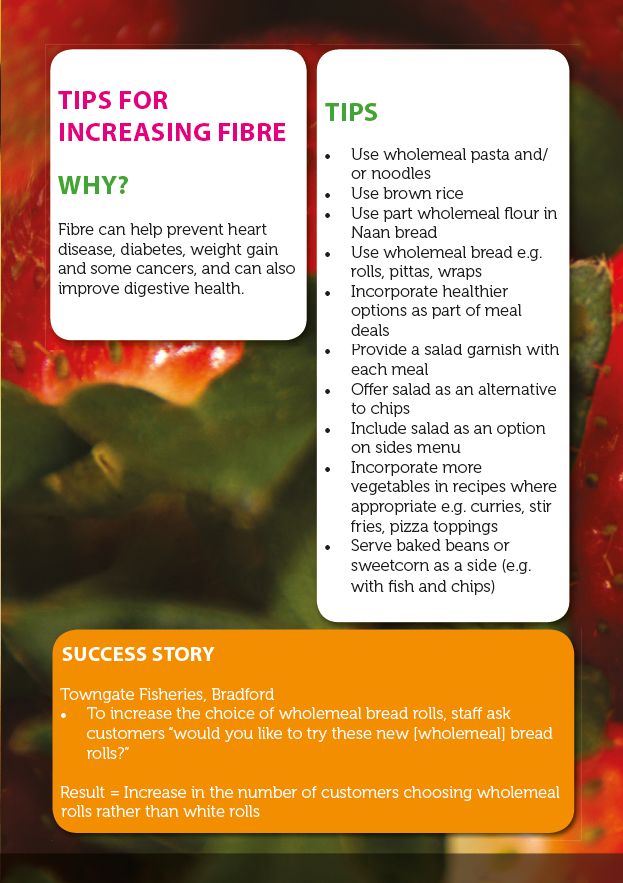

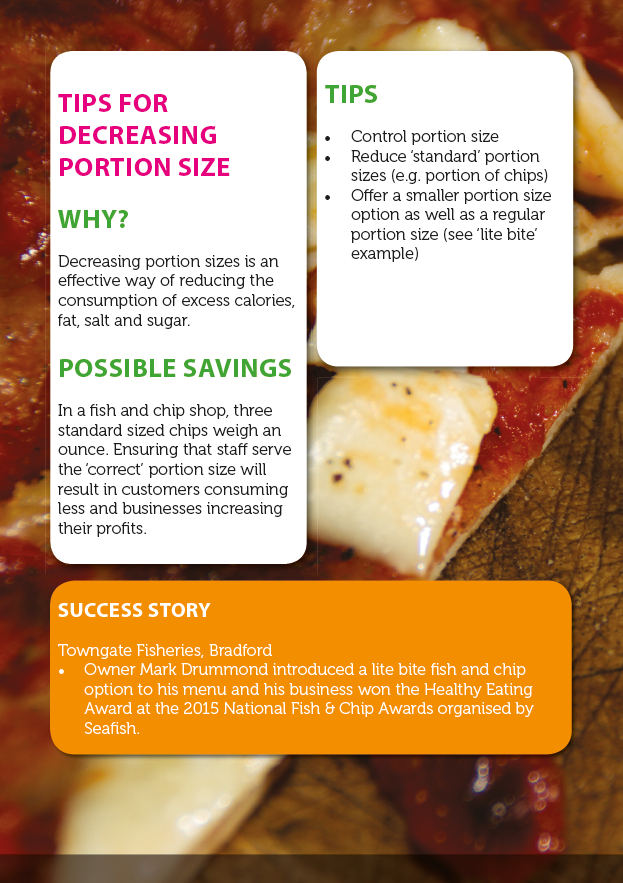


**Supplemental Fig. S4: Takeaway Masterclass Pledge Form**


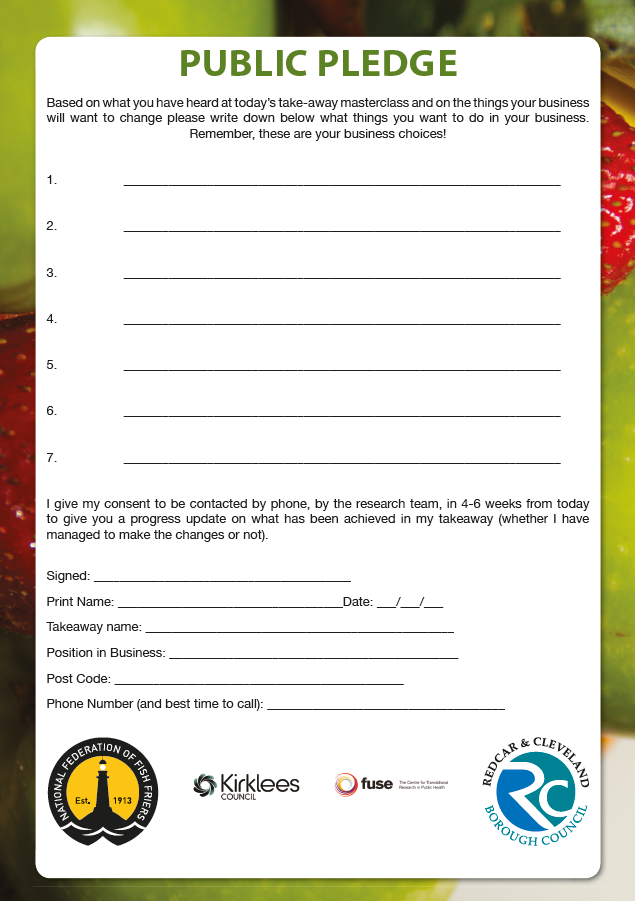

Supplement: Supplementary file 1 [file S1368980019000648sup001.docx]
